# First Molecular Evidence of *Anaplasma bovis* and *Anaplasma phagocytophilum* in Bovine from Central Punjab, Pakistan

**DOI:** 10.3390/pathogens8030155

**Published:** 2019-09-17

**Authors:** Naveed Iqbal, Muhammad Uzair Mukhtar, Jifei Yang, Muhammad Sohail Sajid, Qingli Niu, Guiquan Guan, Zhijie Liu, Hong Yin

**Affiliations:** 1 State Key Laboratory of Veterinary Etiological Biology, Key Laboratory of Veterinary Parasitology of Gansu Province, Lanzhou Veterinary Research Institute, Chinese Academy of Agricultural Sciences, Xujiaping 1, Lanzhou 730046, Gansu, China; 2 Department of Parasitology/One Health Laboratory/Center for Advanced Studies in Agriculture and Food Security, University of Agriculture, Faisalabad 38000, Pakistan; 3 Jiangsu Co-innovation Center for Prevention and Control of Important Animal Infectious Diseases and Zoonoses, Yangzhou 225009, Jiangsu, China

**Keywords:** *Anaplasma bovis*, *Anaplasma phagocytophilum*, nested-PCR, bovine, Pakistan

## Abstract

Obligate intracellular bacteria belonging to the genus *Anaplasma* spp. are responsible for causing a hemolytic disease called anaplasmosis in animals, as well as in humans. This study was aimed at the molecular identification and genetic analysis of responsible causative agents of anaplasmosis beyond those already reported. A survey was performed during July and August 2018 in the Jhang District, Punjab, Pakistan. Four hundred and fifty blood samples from asymptomatic, tick-infested cattle were collected on FTA cards and tested for the *Anaplasma* spp. presence using nested-polymerase chain reaction (PCR) methods. The 16S ribosomal RNA gene sequences generated from the positive samples were used for genetic analysis of *Anaplasma* spp. The nested-PCR results showed the presence of two *Anaplasma* spp. with an overall prevalence rate of 10.44%, where the prevalence of *A. bovis* and *A. phagocytophilum* was 7.78% and 2.66%, respectively. The study portrayed new molecular data on the prevalence of *Anaplasma* spp. in the studied cattle population, indicating a potential threat to the human population as well.

## 1. Introduction

Being an agricultural country, the economy of Pakistan gets a real boost from its livestock industry, with a share of 58.92% in the agriculture sector and a contribution of almost 11.11% during the 2017–2018 economic year, as far as total gross domestic product is concerned. In Pakistan, there are currently 84.9 million heads of buffalo and cattle present, sharing 96.80% of the total milk gross production; whereas 96.03% and 50.56% of the total milk and meat, respectively, is consumed by the human population [http://www.finance.gov.pk/survey_1617.html]. Despite being an integral part of the economy, and despite the fact that a major proportion of the human population of Pakistan is involved in the livestock industry, detailed information regarding disease prevalence and its prevention, management practices and control strategies is lacking.

Because of the developing pathogenicity in farm animals and to a lesser extent in people, among other Rickettsiales, the genus *Anaplasma* demandeds special attention. A variety of the *Anaplasma* species, which is a gram negative bacteria possessing an obligate intracellular nature, is responsible for causing anaplasmosis, a hemolytic tick transmitted disease, in humans and animals. It has a wide distribution in the temperate, subtropical and tropical regions of the world [1]. The disease is continuously becoming a serious concern for the animal breeding system, as the infection puts an additional burden on veterinary care by reducing the body weight of animals, decreasing milk production, and frequently causing abortions leading to death [2,3,4,5].

The *Anaplasma* genus is comprised of six species that exhibit versatility in cell tropism and in the preferential selection of hosts [6,7]. The red blood cells of cattle and wild ruminants are chosen by *A. centrale* and *A. marginale* as a site of infection, while small ruminants presenting the same cells to be infected are encountered by *A. ovis*. *Anaplasma bovis* causing anaplasmosis, targets small mammals and ruminants, which results in the infection of monocytes. Infection is prevalent in different regions of the world with a variable prevalence rate of 3.94 to 39.80% and 9 to 15% in domestic ruminants and wild cervids (Sika deer and Red deer), respectively. The prevalence rate is dependent on the type of species infected and the diagnostic method used [8,9,10,11,12]. Having zoonotic potential, *A. phagocytophilum* preferentially tends to reside and infect neutrophil granulocytes causing granulocytic anaplasmosis in a range of hosts including horses, ruminants, dogs and humans. The organism has been characterized in different regions including Asia, the Americas, Africa and Europe [13,14,15,16,17,18,19]. Age, immune status and the host’s exposure to tick vectors are attributed to its prevalence in different regions [http://www.cdc.gov/anaplasmosis/]. Unique tropism is shown by the *A. platys* bacteria appears in dog platelets and is an etiological agent for infectious canine cyclic thrombocytopenia. Out of six species of the *Anaplasma* genus, five of them specifically look for domestic and wild ruminants to serve as hosts for them [7,13,14,15,16,17,18,19].

From Pakistan, a report is available on the distribution of *A. marginale* and *A. centrale* in cattle and buffaloes from one district of Sindh Province using Giemsa’s stained blood smears, but the study lacks sequence analysis [20]. Whereas some reports using molecular diagnostic approaches have also been made. A study using polymerase chain reaction-restriction fragment length polymorphism (PCR-RFLP) has been carried out reporting one *Anaplasma* spp. (*A. marginale*) only in two provinces of Pakistan [21]. Recently, a PCR-based investigation reported *A. marginale* in the northern areas of Pakistan [22]. To date, however, there is no report on the prevalence of *A. bovis* and *A. phagocytophilum* in bovine from Pakistan, even with the use of microscopic and molecular diagnostic tools.

PCR has been characterized as the gold standard diagnostic approach for anaplasmosis [23] but it has not been used preferentially as a diagnostic tool in most *Anaplasma*-related epidemiological studies in Pakistan. This nested-PCR based study reveals the first 16S ribosomal RNA based evidence of the two *Anaplasma* spp. viz; *A. bovis*, and a zoonotic pathogen, *A. phagocytophilum*, in bovine in the Jhang District, Punjab, Pakistan.

## 2. Results

**Figure 1:**
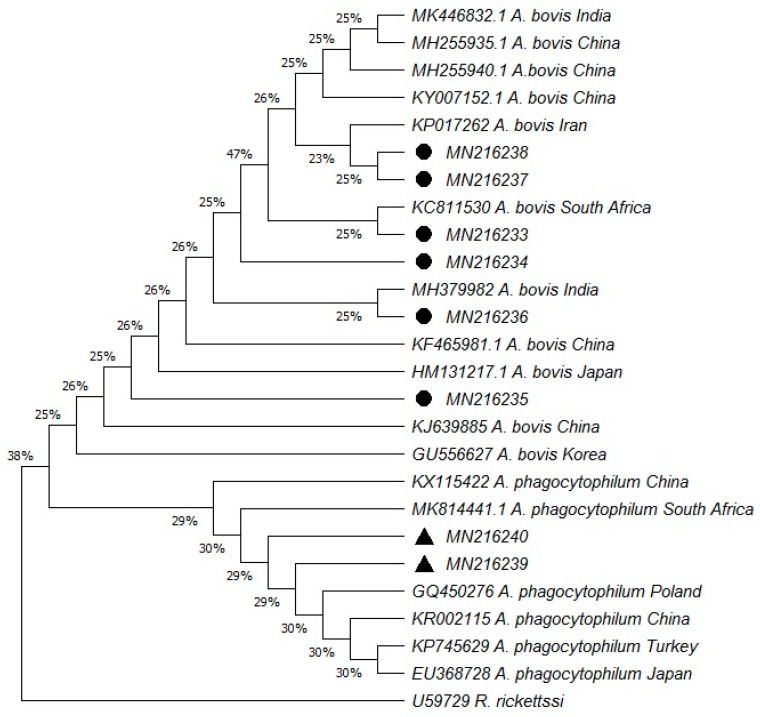
Phylogenetic analysis of the sequences of the 16S rRNA gene using the neighbor-joining method. The optimal tree with the sum of branch length = 1.36582012 is shown. The evolutionary distances were computed using the maximum composite likelihood method. The pathogens identified in the present study are marked in bold, where circles and triangles indicate *A. bovis* and *A. phagocytophilum*, respectively.

Out of 450 bovine blood samples, 47 samples (10.44%) were positive for *Anaplasma* infection. The overall prevalence rate observed for *A. bovis* was higher than that of *A. phagocytphilum*, which was 7.78% and 2.66%, respectively. Sequencing results of the 16S rRNA gene from the positively detected, randomly selected PCR products confirmed the presence of the *Anaplasma* infection in the screened samples that were correctly amplified earlier. The tree constructed on the basis of the 16S rRNA gene explains the phylogenetic relationship (Figure 1).

**Nucleotide sequence accession numbers.** Accession numbers received from NCBI GenBank for 16S rRNA gene sequences of *A. phagocytophilum* are MN216239 and MN216240, while the 16S rRNA gene sequence of *A. bovis* is MN216233-38.

## 3. Discussion

Bovine anaplasmosis caused by different *Anaplasma* spp. is highly endemic in different developing countries [24]. In Pakistan, rural communities commonly fulfill their domestic and commercial needs from small-holder cattle farming systems. However, farmers have also shifted towards commercial dairy farming by adopting modern techniques and importing exotic cattle breeds (*Bos taurus*). While exotic cattle breeds have a greater milk yield potential, they are also at a higher risk of getting ticks and tick borne infections, with a mortality rate more than double of that compared to local breeds (*Bos indicus*).

Ticks have been characterized as the major vectors of *Anaplasma* spp., particularly belonging to the genera *Ixodes*, *Amblyomma*, *Rhipicephalus* and *Dermacentor*. Susceptibility of the animal population towards *Anaplasma* infection is attributed with the distribution and infestation of ticks [25]. Although bovine anaplasmosis exhibits major limitations to the livestock production system, only a few studies provide limited information about bovine anaplasmosis in Pakistan. Most of the exisiting studies rely only on conventional microscopy with low sensitivity and specificity. As far as it could have been ascertained in Pakistan, *A. bovis* has not been detected as an etiological agent of the bovine anaplasmosis and only *A. marginale* has been identified as the major cause of infection from Southern Punjab and Khyber Pakhtunkhwa provinces with prevalence rates of 17% and 18.33% respectively [21,22].

With the objective of defining the spectrum of potential causative agents of bovine anaplasmosis in Pakistan, the designed PCR-based study was successful in diagnosing the *Anaplasma* infection, even in the carrier animals. Use of a PCR tool based on 16S rRNA gene amplification as a preferential method was established for *Anaplasma* spp. detection in carrier animals [26,27,28,29].

This study confers the use of 16S rRNA based nested-PCR in the subclinical diagnosis of the bovine anaplasmosis as it revealed the presence of *A. bovis* and *A. phagocytophilum* in apparently asymptomatic animals with an overall prevalence rate of 10.44%. The obtained sequences showed 98–100% identity to the *Anaplasma* reference sequences. The retrieved sequences of *A. bovis* and *A. phagocytophilum* isolates using BLAST query for phylogenetic analysis presented themselves to be highly homologous with NCBI reference sequences. Phylogenetic analysis of *A. bovis* revealed maximum identity with the sequences previously reported in China, India, Iran, Japan and South Africa. While sequences from *A. phagocytophilum* were obtained from positive samples and were seen to be closely related with the sequences previously reported from South Africa and Poland (Figure 1).

Our findings were dissimilar to an investigation that reported 61% prevalence of *Anaplasma* spp. in Karachi and its adjacent areas [25]. The difference of experimental outcomes regarding *Anaplasma* infection in the two different studies is presumably attributable to the environmental compatibility of Karachi, a coastal city with high relative humidity and moderate climate which supports tick infestation and the different tools used for the pathogen detection [30]. By using conventional microscopy, *Anaplasma* was detected with a very high prevalence rate of 80%, but with no specific differential distribution and evidence of *A. bovis* and *A. phagocytophilum* [31]. Another study made in the neighboring country, Iran, reported 22.22% prevalence rate of *Anaplasma* infection in the cattle population, without species differentiation [32]. Likewise, an investigation claimed the presence of only *A. marginale* with 68.75% prevalence in bovine in Punjab State of India [33]. *Anaplasma bovis* and *A. phagocytophilum* have been identified in Xinjiang, China in cattle with 4.80% and 6.40% prevalence, respectively [11]. In contrast, other researchers described the prevalence of *Anaplasma* infection ranging between 4–12% without establishing *A. bovis* and *A. phagocytophilum* as potential pathogens for the infection in Pakistan, where only the conventional tools for detection were used [34,35].

## 4. Methods

### 4.1. Sample Collection

In the present study, blood samples were collected randomly on Whatman FTA^TM^ Classic Cards from 450 tick-infested but asymptomatic cattle during July and August 2018 from Jhang District in Punjab, Pakistan. Blood was collected from the jugular vein using 10 ml disposable syringes and transferred to the Whatman® FTA cards (GE Healthcare Limited, Buckinghamshire HP7 9NA, UK) and allowed to air-dry. Sample collection and animal treatments complied with the Animal Ethics Procedures and Guidelines and was approved by the Animal Ethics Committee of the Punjab Livestock Department, Pakistan. The FTA cards were shipped to Lanzhou Veterinary Research Institute at the Chinese Academy of Agricultural Sciences Lanzhou, China for further processing.

### 4.2. DNA Extraction

Genomic DNA was extracted from the FTA cards using the QIAamp DNA Mini Kit (Qiagen, Hilden, Germany) according to the manufacturer’s instructions. The DNA concentration was determined with a Nano-Drop 2000 spectrophotometer (Nanodrop Technologies®, Wilmington, DE, USA). DNA was stored at −20 °C until further analysis.

### 4.3. PCR Amplification

Nested PCR was carried out to detect *Anaplasma* infection from the collected bovine samples. During the first round, genomic DNA from field blood samples was amplified using the primers EE1 and EE2 [36]. The PCR products were used as templates for the second round using the *A. bovis* specific primers AB1f and AB1r, which generate a product of 551 bp, and the *A. phagocytophilum* specific primers SSAP2f and SSAP2r, which generate a product of 641 bp [13]. The reactions were performed in a final volume of 50 µL, containing 1.0 mM of each primer, 5 µL of PCR buffer, 4 µL of deoxynucleoside triphosphates, 0.25 µL of TaKaRa Taq (5 µ/mL) (TaKaRa, China), and 1 µL of DNA sample. Reactions were conducted in an automated DNA C1000 Thermal Cycler (Bio-Rad, Beijing, China). For the EE1 and EE2 primers, the cycling conditions were denaturation for 4 min at 94 °C, followed by 94 °C for 30 s, 62 °C for 30 s, and 72 °C for 30 s. The annealing temperature (62 °C) was stepped down four times by 2 °C every two cycles. The final annealing temperature used was 54 °C for 28 cycles, followed by a final extension for 5 min at 72 °C. For the nested PCR, 2 µL of the product from the first amplification was used for amplification with specific primers; the amplification consisted of 40 cycles, each of 1 min at 94 °C, 1 min at 55 °C, and 1 min at 72 °C. Cattle genomic DNA and distilled water were used as negative and blank controls, respectively. The PCR products were subjected to electrophoresis on 1% agarose gel containing 0.5 g/ml ethidium bromide and visualized under UV light.

#### DNA Sequencing and Data Analysis

Positive PCR products amplified by primers SSAP2f/2r and AB1f/AB1r were excised from the gel and purified using the AxyPrep DNA Gel Extraction Kit (Axygen, USA). The DNA fragments were cloned into pGEM-T vector (Promega, Madison, WI). *Escherichia coli* Trans 5α (TaKaRa, China) was transformed and plasmid DNA from the selected clones was identified using PCR with the set of primers T7 (5’-TAATACGACTCACTATAG GG-3’) and SP6 (5’-ATTTAGGTGACACTATAG-3’) to verify the presence of correct inserts in selected clones and then sequenced by Sangon Biotech Company (Shanghai, China). The obtained sequences were analyzed by a BLAST search in GenBank for determining the accuracy of the PCR method.

### 4.4. Phylogenetic Analysis

For genotyping, obtained sequences of *A. phagocytophilum* and *A. bovis* were aligned using the MegAlign component of the DNAStar software program (Version 4.0 DNAStar, Madison, USA). After alignment with related *Anaplasma* spp. 16S rDNA sequences retrieved from GenBank, parts of the cloning vector region were removed manually. The resulting sequences were then submitted to the GenBank database. A phylogenetic tree was generated based on the cloned sequences and the related *Anaplasma* spp. 16S rDNA sequences in GenBank by using the neighbor-joining method [37].

## 5. Conclusions

The present study provides the first evidence of *A. bovis* and *A. phagocytophilum* as potential causative agents of bovine anaplasmosis in Pakistan; of which the latter alarms for its own health significance. A comprehensive molecular epidemiological investigation is required for appropriate disease mapping in the country which can help devise control strategies for ticks and tick-transmitted diseases of livestock and public health significance. 

## Data Availability

Sequences submitted in the GenBank database under accession numbers are as follows: *A. phagocytophilum*: MN216239 and MN216240; and *A. bovis*: MN216233, MN216234, MN216235, MN216236, MN216237 and MN216238.

## Abbreviations

PCR	polymerase chain reaction
DNA	deoxyribonucleic acid
